# Macrophage polarization in gynecologic malignancies: key signaling pathways and clinical perspectives

**DOI:** 10.3389/fimmu.2026.1884478

**Published:** 2026-06-30

**Authors:** Qian He, Yali Chen

**Affiliations:** 1 Department of Obstetrics and Gynecology, West China Second University Hospital, Sichuan University, Chengdu, Sichuan, China; 2 Key Laboratory of Birth Defects and Related Diseases of Women and Children (Sichuan University), Ministry of Education, Chengdu, Sichuan, China

**Keywords:** gynecological malignancies, NDDSs, polarization, signaling pathways, TAMs

## Abstract

Gynecological malignancies represent a significant global health burden for women worldwide. Tumor-associated macrophages (TAMs), as a critical component of the immune system, play a pivotal role within the tumor microenvironment (TME). Modulating signaling pathways in the TME can promote the polarization of TAMs toward the M1 phenotype, thereby enhancing anti-tumor effects and suppressing tumor growth and metastasis. The rapid advancement of nano-drug delivery systems (NDDSs) has expanded the potential for clinical translation of immunotherapies targeting TAMs. Our review provides an overview of the latest research progress on TAMs polarization-related signaling pathways and the use of NDDSs for targeted TAMs therapy in gynecological malignancies. Concurrently, the challenges encountered during the clinical translation of these therapeutic measures and future directions are discussed, aiming to provide novel insights for the immunotherapy of gynecological malignancies.

## 1 Introduction

Gynecological malignancies primarily include ovarian cancer (OC), cervical cancer (CC), and endometrial cancer (EC). According to the latest data from the International Agency for Research on Cancer (IARC), the incidence of female reproductive system malignancies has shown an overall increasing trend among newly diagnosed cases of malignant tumors globally (1). As a complex ecosystem, the tumor microenvironment (TME) encompasses cellular elements, including tumor and immune cells, as well as acellular elements like signaling molecules (2). There is an inextricable link between the TME and immunotherapy, where it acts as both the critical foundation for therapeutic efficacy and a major barrier leading to treatment resistance. Tumor immunotherapy is a class of therapeutic approaches that harnesses the body’s own immune system to recognize, attack, and eliminate cancer cells (3). Over the past decades, antitumor immunotherapy has achieved significant breakthroughs, demonstrating unprecedented efficacy in the field of gynecological malignancies (4–6). At present, T-cell function-modulating therapies, represented by immune checkpoint inhibitors, have established a crucial role in the treatment of gynecological malignancies. In contrast, drugs targeting the regulation of TAMs remain in the early stages of research and exploration.

TAMs represent the most abundant immune cell population within the TME, accounting for up to 50% of the cellular composition in some solid tumors (7). TAMs exhibit remarkable plasticity and heterogeneity, playing a central role in regulating tumor-associated inflammation. Within the TME, TAMs are primarily classified into M1 and M2 types based on their functional characteristics and phenotypes, and the M2 phenotype can be further subdivided into M2a, M2b, M2c, and M2d subsets (8). M2-type macrophages secrete immunosuppressive factors that can suppress the pro-inflammatory and anti-tumor functions of M1-type macrophages, thereby weakening the host immune response and promoting tumor progression. Consequently, M2-type macrophages typically predominate in the TME (9). However, recent single-cell RNA sequencing (scRNA-seq) analyses have revealed the complexity of macrophage subsets within the TME, which extends beyond the simplistic M1/M2 dichotomy (10). Therapeutic strategies targeting TAMs aim to deplete their numbers, reprogram their functions, and employ other regulatory approaches to reverse the immunosuppressive state within the TME, thereby effectively inhibiting tumor progression (11–13). TAM polarization denotes the process wherein macrophages acquire divergent functional and phenotypic characteristics under the influence of specific TME cues. Such polarization is orchestrated by a complex, multi-level regulatory network involving transcription factor modulation (14), gene editing (15), metabolic reprogramming (16), and epigenetic modifications (17). However, nanoparticle (NP)-based nanomedicine delivery systems have opened a new pathway for the clinical translation of macrophage-targeted immunotherapy (18).

**Figure 1:**
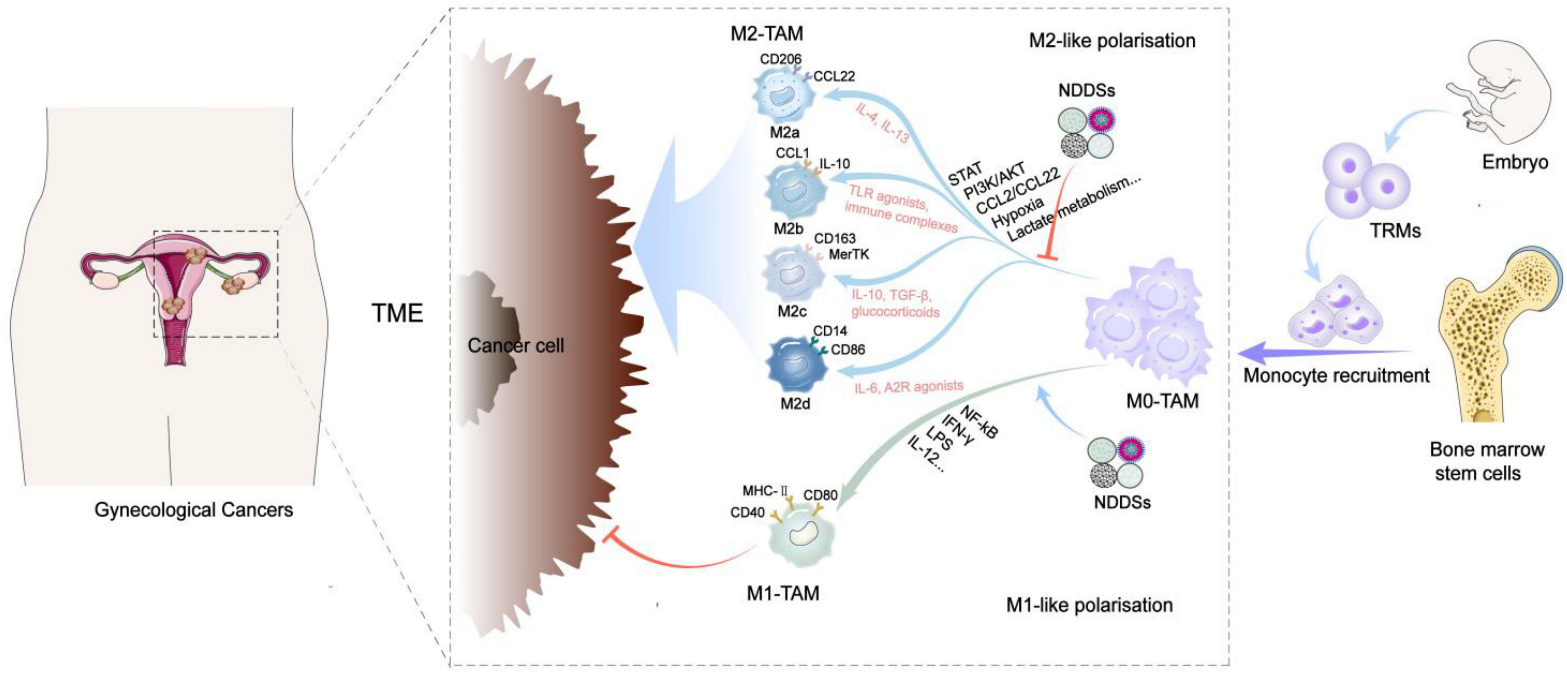
Origin and polarization pathways of TAMs in gynecologic malignancies. Monocytes in the TME are driven toward M1 or M2-type states by specific biomolecules or NDDSs.

Therefore, in-depth investigation into the signaling pathways of TAM polarization during tumor progression and treatment holds significant importance for the development of novel therapeutic strategies. Our review primarily focuses on the mechanisms underlying TAM polarization in gynecological malignancies and the latest advancements in NDDSs (**Figure 1**). It aims to provide further insights for drug development and clinical application prospects.

## 2 Origin and polarization of TAMs

**Figure 2:**
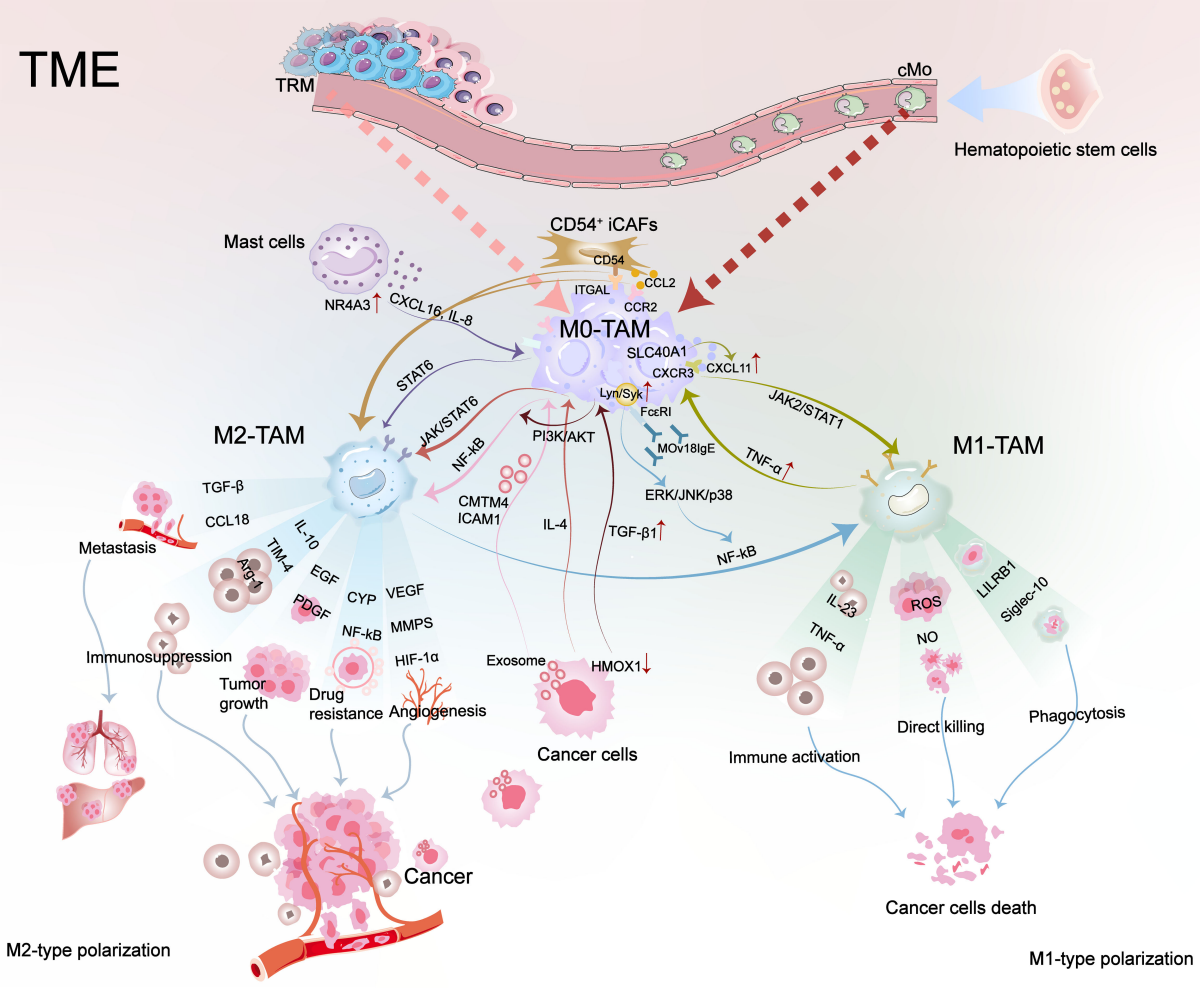
M1-type or M2-type macrophages and their respective functions. Notably, M1-type TAMs are characterized by immune activation, killing tumor cell, and phagocytosis. In contrast, M2-type TAMs contribute to tumor immunosuppression, growth, drug resistance, angiogenesis, and metastasis.

Macrophages are immune cells belonging to the mononuclear phagocyte system and play a crucial role in immune defense, surveillance, and homeostasis (19). Under steady-state conditions, macrophages originate from two distinct hematopoietic pathways. One pathway is derived from adult bone marrow hematopoiesis, which gives rise to circulating monocytes that differentiate into monocyte-derived macrophages (Mo-Macs). The other originates from embryonic precursor cells, giving rise to tissue-resident macrophages (TRMs) (20). TAMs are derived predominantly from circulating monocytes and TRMs, showing significant heterogeneity in proportion and phenotype throughout tumor development. During the early tumor stages, TRMs promote tumorigenesis by mediating inflammatory responses. However, as the tumor progresses, TAMs in the core region are predominantly derived from Mo-Macs, whereas TRMs are primarily distributed in the tumor periphery (21). Furthermore, tumor-associated macrophages (TAMs) represent the most abundant immune cell population within solid tumors and exhibit a dual functionality (**Figure 2**). This paradoxical characteristic stems from their high degree of heterogeneity, which is shaped by a combination of factors such as developmental origins, transcriptional programs, and signals from the TME (11). With continued tumor progression, an acidic and hypoxic microenvironment emerges, driving macrophage polarization toward an M2 phenotype through distinct signaling pathways, thereby facilitating accelerated tumor growth and metastasis (9). Polarization of TAMs is defined as their differential activation in the TME in response to distinct signals, broadly categorized into the M1 and M2 phenotypes. Among these, the M1 phenotype exerts pro-inflammatory and anti-tumor effects, while the M2 phenotype promotes tumorigenesis and suppresses inflammatory responses. In tumor tissues, a substantial proportion of TAMs polarize into the M2 phenotype, which shields tumors from immune surveillance and consequently promotes tumor initiation, invasion, and metastasis (22). With the advancement of scRNA-seq, an increasing number of functional subtypes of TAMs have been identified (23, 24). In a 2024 review, researchers proposed classifying TAMs into inflammatory TAMs (Inf-TAM), angiogenic TAMs (Ang-TAM), regulatory TAMs (Reg-TAM), interferon-mediated regulatory TAMs (Inf-Reg TAM), immunostimulatory macrophages, and CD169+ macrophages in lymph nodes (25). However, classification criteria vary among researchers. In actual scRNA-seq data, different TAM subsets may display overlapping gene signatures or graded expression patterns, rendering definitive discrimination solely by transcriptional profiles difficult. It is hypothesized that TAMs may undergo a continuous phenotypic transition between subsets within the specific TME. Current studies have demonstrated that targeting TAMs can improve patient resistance to immune checkpoint inhibitor therapy and lead to superior therapeutic outcomes (26, 27). Consequently, TAMs represent a promising therapeutic target in tumor immunotherapy. Exploring the signaling pathways that drive their polarization in gynecological malignancies holds significant clinical potential.

## 3 OC and TAM polarization

OC has a very poor prognosis, and its unfavorable outcome is closely associated with the complex TME (28, 29). In the current clinical context of immunotherapy, the majority of OSs are classified as immunologically “cold tumors, “ characterized typically by sparse infiltration of cytotoxic T cells and substantial enrichment of immunosuppressive cells, such as regulatory T cells (Tregs), myeloid-derived suppressor cells (MDSCs), and M2-type macrophages (30, 31). Furthermore, in patient samples of OC, scRNA-seq revealed that TAMs accounted for approximately 30% of cells in primary tumors, while this proportion increased to about 45% in drug-resistant tumors (32). Therefore, targeting and activating TAMs to repolarize them into a pro-inflammatory, anti-tumor state represents a crucial therapeutic strategy for OC. In samples from OC metastasis and dissemination, as well as in animal models, recent studies have identified signaling pathways that regulate the polarization of TAMs toward the M2 phenotypes. Within the TME, tumor-associated mast cells (MCs) expressing high levels of NR4A3 secrete elevated levels of CXCL16 and IL-8. These cytokines activate the STAT6 signaling pathway, driving macrophage polarization toward an M2-like phenotype, thereby facilitating metastasis in OC (32). Downregulation of HMOX1 expression within tumor epithelial cells drives M2-type macrophage polarization via the transforming growth factor-β1 (TGF-β1)/PI3K/AKT/NF-κB (p65) signaling pathway (33). Therefore, constructing an immunotherapy response prediction model based on NR4A3, HMOX1, and TGF-β1 holds potential clinical application value. OC cell-derived exosomal CMTM4 is internalized by macrophages, activating the ICAM1-dependent NF-κB signaling pathway to promote M2-type macrophage polarization and immunosuppressive functions, thereby facilitating OC progression and immune escape (34). Meanwhile, interleukin-4 (IL-4) produced by cancer cells as well as Makorin-2 (MKRN2) deficiency in the TME also contribute to M2-type macrophage polarization (35, 36). Blocking the signaling pathways associated with the above-mentioned M2-type macrophage polarization can inhibit the progression of OC. SLC40A1 promotes M1-type macrophage polarization by regulating CXCL11 secretion to activate the JAK2-STAT1 signaling pathway, which enhances TNF−α production in macrophages and thereby forms a positive feedback loop that further upregulates SLC40A1 expression (37). The OC TME drives macrophage polarization toward the immunosuppressive M2c phenotype *via* the IL−10/JAK–STAT3 signaling pathway; conversely, MOv18 IgE activates the Lyn/Syk–MAPK–NF−κB pathway through FcϵRI cross−linking, thereby repolarizing M2-type macrophages to a pro−inflammatory phenotype, reversing immunosuppression, and enhancing anti−tumor immunity (31). *In vitro* studies demonstrate that macrophages treated with anti-STAB1 exhibit significantly higher TNF−α and CD11c expression, as well as reduced PD−L1 expression, confirming that anti−STAB1 antibodies reprogram TAMs toward an M1 phenotype (38). Blocking sialic acid-binding immunoglobulin-like lectin-9 (Siglec-9) inhibits the phosphorylation of phosphatase SHP-1, ultimately repolarizing TAMs to the M1 phenotype (39).

At present, chemotherapy is still a crucial approach for treating OC, and TAMs play a vital role in the drug resistance that emerges during chemotherapy. In the paclitaxel-treated mouse OC model, it was observed that NOTCH2 on the tumor cell surface was upregulated at the translational level, inducing the secretion of cytokines such as colony stimulating factor 1 (CSF1) and Interleukin-1 beta (IL-1β), which recruited more M2-type macrophages (JAG1^+^ macrophages) to establish a positive feedback loop that enhances drug resistance (40). In addition, peripheral serotonin promotes the formation of M2-type macrophages (HTR7^+^ macrophages), inducing them to secrete extracellular vesicles containing inositol metabolic enzymes (EVs). This elevates nuclear inositol tetrakisphosphate (IP4) levels in cancer cells, enhances MRE11-mediated homologous recombination repair, and thereby drives chemotherapy resistance in OC (32). Research into the chemoresistance mechanisms involving M2-type macrophage polarization has expanded novel perspectives and avenues for enhancing chemotherapy efficacy.

Collectively, emerging evidence delineates the phenotypic plasticity of OC TAMs, implicating key signaling regulators such as NR4A3, STAT6, SLC40A1, and NOTCH2. Therapeutic strategies designed to modulate macrophage polarization—specifically by targeting critical cytokines or downstream effectors—hold significant promise in synergizing with chemotherapy, anti-angiogenic agents, and immune checkpoint inhibitors. These approaches offer a potential breakthrough in overcoming current therapeutic limitations and improving patient outcomes in OC.

## 4 CC and TAM polarization

Cervical cancer (CC) is the fourth most frequently diagnosed cancer and the fourth leading cause of cancer-related deaths among women globally, with its incidence and mortality rates second only to breast cancer (1). Chronic inflammation induced by high-risk HPV is considered an important risk factor for CC (41). During the progression from chronic cervicitis to invasive CC, the expression of macrophages and neovascularization in the TME increases synchronously (42). In HPV-positive cervical cancer samples, the proportion of myeloid cells, including SPP1^+^ macrophages, is higher than that in HPV-negative samples, suggesting that HPV infection may drive the recruitment and polarization of these immunosuppressive myeloid cells. Furthermore, animal studies have demonstrated that SPP1^+^ macrophages shape an immunosuppressive microenvironment and promote tumor progression via the SPP1–CD44 axis (43). In cellular and mouse models, CD54^+^ iCAFs recruit monocytes and promote their polarization toward an ITGAL^+^ M2-type macrophages *via* the CCL2-mediated signaling axis (44).

Hypoxic and acidic TME are ubiquitous in solid tumors, which not only promote angiogenesis but also lead to therapeutic resistance and immune escape. Under hypoxic conditions within the CC TME, TAMs promote the formation of the lymphatic vessels encapsulated and tumor cell metastasis by secreting IL-10 to activate STAT3 signaling, upregulating Sp1 expression, and inducing CCL1 overexpression in lymphatic endothelial cells to recruit additional macrophages and tumor cells (45). Inhibitor of β-catenin and TCF (ICAT) promotes H3K18la modification in TAMs by upregulating lactate levels in the TME, thereby inducing M2-type polarization (46). Recent studies have found that signals derived from CC cells themselves participate in the regulation of TAM polarization to facilitate immunosuppression and distant metastasis. Based on bioinformatic analysis and *in vitro* cellular experiments, high expression of the ferroptosis-related gene GCH1 suppresses M2-type macrophage infiltration and promotes M1-type macrophage polarization *via* activation of the PI3K/AKT/mTOR signaling pathway, ultimately improving the prognosis of patients (47). *In vitro* studies confirmed that CCL22 secreted by CC TAMs drives M2a polarization *via* autocrine CD206 upregulation (48). Furthermore*, in vitro* and *in vivo* experiments verified that CC cells secrete CMTM6-laden exosomes. These exosomes activate the mTOR signaling pathway to induce M2a macrophage polarization and ultimately establish an immunosuppressive microenvironment (49). Researchers have explicitly demonstrated that blocking the intrinsic signaling or metabolic pathways of TAMs inhibits M2 polarization and enhances their antitumor activity. *In vitro* cellular models, SEMA6B expressed in TAMs inhibits M2 polarization and migration *via* suppression of AKT phosphorylation (50). CD74 inhibits phagocytosis and induces M2 polarization; blocking CD74 derived from TAMs can enhance the efficacy of neoadjuvant therapy (51). Targeting macrophage-associated HK3 combined with immune checkpoint blockade exerts a synergistic effect to enhance antitumor immunity (52).

In the treatment of cervical cancer, TAM-mediated drug resistance and metastasis remain clinically challenging problems. Existing therapeutic modalities often struggle to precisely intervene in their complex pro-tumorigenic signaling networks (e.g., CCL22, SEMA6B, CD74 pathways), and systemic inhibition of macrophages may compromise normal immune function. Future efforts should focus on exploring combination intervention strategies based on molecular subtyping and developing local targeting technologies. These measures aim to effectively reverse the pro-tumorigenic functions of macrophages while maintaining the overall immune balance of the body, thereby overcoming current therapeutic bottlenecks.

## 5 EC and TAM polarization

EC is one of the most common gynecologic malignancies, with its global incidence continuing to rise (6). EC, once linked to estrogen-driven proliferation, is now confronted with an increasing prevalence of non-endometrioid subtypes associated with obesity and metabolic dysfunction (53). TAMs play a significant role in the progression of EC, a phenomenon characterized by certain racial disparities (54). In early-stage EC tissue samples, researchers found that epithelial subsets interact with macrophages *via* the CD47-HCK-LGALS9 axis to establish an immunosuppressive microenvironment (55). Studies utilizing tissue samples and *in vitro* models have also yielded new findings. Notably, elevated PAX8 in tumor cells drives the polarization of TAMs toward the M2 phenotype *via* paracrine signaling—characterized by the inhibition of IL-1A, IL-1B, IL-11 and the induction of TGF-β1, TGF-β2—thereby forming an immunosuppressive microenvironment that mediates tumor immune escape and ethnicity-related poor prognosis (54). Furthermore, ARNTL reprograms glycolytic metabolism in EC cells *via* the ARNTL-INO80-DHX15 axis, thereby driving macrophage polarization toward the M2 phenotype and mediating tumor immune escape (56). EC organoid models confirmed that transcription factor ERRα modulates M2-type macrophage polarization and infiltration through the PTPMT1-CCL2 axis (57). Lactate derived from EC cells promotes macrophage M2-type polarization and enhances epithelial-mesenchymal transition and angiogenesis, consequently increasing the aggressiveness and metastatic potential (58). Mechanistic studies confirmed that the above biological behaviors of EC are regulated by the DNMT1-NHE7 axis (59). In addition to the regulation of macrophages by relevant signals expressed by tumor cells, macrophages also positively feedback to tumor cells through certain signals. For instance, KYNU secreted by M2-type macrophages modulates the malignant biological behaviors of EC via the SOD2-mtROS-ERO1α axis and the endoplasmic reticulum unfolded protein response (UPR) pathway (60).

EC is a malignant tumor in women closely associated with metabolic dysregulation. Most EC patients respond poorly to immunotherapy, highlighting the necessity of identifying new therapeutic targets at the intersection of metabolism and immune regulation. Based on bioinformatics analysis and animal experiments, signaling targets regulating macrophage polarization have emerged as a new direction for immunotherapy.

## 6 Application of NDDSs targeting TAMs in gynecological malignancies

Cancer remains one of the most intricate and formidable challenges in modern medicine, propelling extensive scientific research worldwide. Certain tumor therapeutics are frequently limited in clinical utility due to poor targeting, restricted efficacy, and severe side effects associated with systemic administration. NDDSs, characterized by precise targeting and controllable drug release, not only mitigate adverse effects but also significantly ameliorate patient prognosis, revealing the potential to revolutionize traditional cancer therapy. As insights into the TME deepen, the targeting focus of NDDSs has expanded from cancer cells to stromal components, including tumor-associated immune cells and fibroblasts.

Significant progress has also been made in targeting TAMs in gynecological malignancies based on NDDSs. In a mouse model of peritoneal metastasis of OC, researchers found that rigid inorganic SiNPs exhibit specific targeting towards TAMs (61). Potent immunostimulatory factor IL-12 holds great promise; however, its clinical application is hindered by severe dose-limiting toxicity. NPs formed by immobilizing IL-12 on the surface of liposomes enable more targeted drug delivery to tumor tissues, effectively resolving the conflict between the toxicity of systemic IL-12 administration and insufficient local concentration in OC (62). In CC and orthotopic OC models, GAZn-PEG NPs combined with HIFU inhibited M2-type macrophages and increased M1-type macrophages (63). Furthermore, a multifunctional smart NDDS, H/D@FA-CaP-NPs, can downregulate the BACH1/CD47 axis to overcome chemoresistance in OC.

Despite extensive research on the development of novel and efficient nanomedicines, only a few NDDSs have been successfully translated into new therapeutic drugs to date. The successful translation of NDDSs into clinical applications faces numerous challenges, including complex interactions between NPs and biological systems, heterogeneous tumor penetration and retention, safety issues, difficulties in precise targeting, and technical barriers to large-scale production, all of which urgently need to be addressed (64). Specifically, the enhanced permeability and retention (EPR) effect in human tumors exhibits substantial heterogeneity, resulting in an average tumor accumulation of NPs of less than 1% of the administered dose. Furthermore, non-specific sequestration by the liver and spleen causes severe toxicity and adverse effects (65). Concurrently, patients exhibit significant heterogeneity in responses to nanomedicines, and suitable biomarkers for accurate prediction of clinical outcomes remain lacking. Currently, our understanding of the long-term immune memory induced by nanocarriers and their associated chronic toxicity remains insufficient, while their biocompatibility requires further evaluation. The clinical application of NDDSs faces challenges in batch quality control and regulatory approval. Meanwhile, existing preclinical models are limited in their ability to replicate the complex human TME and immune responses (66). Additionally, the heterogeneity of surface markers on different immune cells demands higher design flexibility for nanocarriers. With the continuous integration of novel screening technologies, artificial intelligence (AI), and more sample data, the design of cancer NDDSs is constantly being optimized. By combining these multidisciplinary achievements, scientists are expected to develop more efficient and personalized targeted therapies, bringing greater clinical benefits to cancer patients.

## 7 Prospects and outlooks

Gynecological malignancies refer to tumorous diseases occurring within the female reproductive system, with an increasing incidence year by year, imposing a substantial medical burden (67). In recent years, anti-tumor immunotherapy has made breakthrough progress in gynecological oncology. Its core strategy is to enhance anti-tumor immune responses by reactivating innate and adaptive immunity (68). As an important component of the immune system, macrophages play a pivotal role in the tumor microenvironment. Abundant evidence indicates that tumor-associated macrophages can simultaneously express both M1- and M2-related genes, and their gene expression manifests as a continuous dynamic spectrum, where genetic and molecular characteristics are not mutually exclusive (69). Leveraging emerging detection technologies and artificial AI to identify functionally distinct TAM subsets and polarization-related signaling targets will provide selective and universal targets for precise TAM-targeted immunotherapy in gynecological malignancies. Therefore, how to precisely regulate the function of TAMs and shift them from a “pro-tumor” state to an “anti-tumor” state is an important research direction in tumor immunotherapy. TAMs possess high heterogeneity, and their phenotypes and functions are subject to multidimensional precise regulation by tumor type, developmental stage, and signals within the microenvironment (10). Multiple metabolic processes in the TME play a vital role in modulating the phenotypes of TAMs and sustaining their biological functions (70). Metabolic reprogramming in TAMs is not driven by a single pathway; instead, it arises from the coordinated crosstalk among glucose, lipid, and amino acid metabolic networks (71). In gynecological malignancies, accumulating evidence has demonstrated that acidic microenvironment, ferroptosis and hypoxia facilitate M2-type macrophage polarization (33, 45, 46). Moreover, lipid metabolism contributes to modulating TAM reprogramming in the OC microenvironment (72). Modulating the availability of key metabolites in the TME can directly regulate macrophage polarization and function. Therefore, targeting the metabolism-TAM axis provides novel strategies and targets for immunotherapy against gynecological malignancies. Recent advances in single-cell and spatial omics have facilitated the characterization of the molecular profiles and regulatory mechanisms underlying diverse TAM subpopulations (11). These discoveries undermine the conventional classification paradigm, highlighting the need to construct a multi-dimensional classification system.

Based on the aforementioned elaboration on TAM polarization signaling, it is evident that TGF−β, STAT, and PI3K/AKT serve as common core pathways driving TAM polarization toward the M2 phenotype in OC, CC, and EC. Furthermore, CCL2/CCL22, IL−10, hypoxia, lactate metabolism, and ferroptosis−related signals are jointly involved in regulating the protumoral M2 phenotype. Moreover, the regulatory mechanisms of TAM polarization display distinct disease−specific features among the three malignancies. OC is characterized by NR4A3/STAT6, NOTCH2, and SLC40A1/JAK2/STAT1 pathways, which mediate chemotherapy resistance; CC is modulated by SPP1, SEMA6B, and CD74 to promote lymph node metastasis; whereas EC is predominantly governed by PAX8, ARNTL, and ERRα, which are closely associated with metabolic disorders and ethnic disparities. In gynecologic malignancies, small-molecule drugs targeting signaling pathways exert anti-tumor effects by modulating TAM phenotypes, and multiple such agents are currently in various phases of clinical research (**Table 1**). Oral and intravenous administration routes result in systemic drug distribution, which predisposes to toxic side effects in non-target organs. NDDSs effectively overcome the limitations of traditional administration routes—such as low bioavailability, drug degradation, and off-target toxicity—by achieving targeted delivery and controlled release (65). NDDSs targeting TAMs in gynecologic malignancies has been extensively investigated and holds substantial promise for clinical application (66, 73). Nevertheless, the successful clinical translation of NDDSs currently faces numerous challenges, including complex interactions of NPs within biological systems, heterogeneous tumor penetration and retention, safety concerns, and technical barriers to large-scale manufacturing, which urgently need to be addressed.

**Table 1:** Clinical trials on targeting macrophages for the treatment of gynecologic malignancies.

Drug name	Gynecologic malignancies	Signaling target	Trial phase	ClinicalTrials.gov ID	Article DOI
ALX148	OC	CD47	Phase II	NCT05467670	/
Sargramostim	OC	GM-CSF	Phase II	NCT00157573	DOI:10.1016/j.ygyno.2009.10.075
Vigil	OC	GM-CSF	Phase II	NCT01309230	DOI:10.1016/j.ygyno.2016.09.018 DOI:10.1016/j.gore.2020.100648 DOI:10.3390/vaccines9080894
Sargramostim+Abraxane	OC	GM-CSF	Phase II	NCT00466960	DO:10.1200/jco.2015.33.15_suppl.5580
Sargramostim+pNGVL3-hICD vaccine	OC	GM-CSF	Phase I	NCT00436254	DOI:10.1001/jamaoncol.2022.5143
MCS110 + PDR001	EC	CSF-1	Phase II	NCT02807844	DOI:10.36401/JIPO-23-16
CT-0508	OC、CC、EC	HER2	Phase I	NCT04660929	DOI:10.1038/s41587-020-0462-y DOI:10.1038/s41591-025-03495-z DOI:10.1016/j.it.2022.10.002
Zoledronic Acid	OC	IL-10, TGF-β, VEGF	Early Phase 1	NCT05053750	/

Tumors are characterized by heterogeneity and a complex immunosuppressive microenvironment, which renders monotherapy prone to drug resistance or unsatisfactory therapeutic efficacy. Combination therapy exerts synergistic effects via multiple targets and mechanisms, thereby overcoming drug resistance and enhancing anti-tumor efficacy. Recent studies have highlighted that the combination of TAM modulators with immune checkpoint inhibitors (ICIs) remodels the immunosuppressive TME, thereby restoring T-cell cytotoxicity and exerting synergistic antitumor effects (74). Similar synergistic effects have likewise been observed when TAM-targeted therapy is combined with chemotherapy and radiotherapy (32, 75). However, combination therapy may elevate the risk of toxicity, necessitating optimization of drug dosages and administration schedules. Tumor heterogeneity and clonal evolution complicate drug resistance mechanisms, underscoring the need to develop more precise biomarkers for treatment guidance. Integrating AI and big data analytics is critical to implement personalized combination therapy and improve therapeutic efficacy and safety.

Due to the critical involvement of TAMs in fostering tumor progression and metastasis while inhibiting immune responses in gynecologic cancers, TAM-targeted strategies have become a focal point of research. NDDSs further enhance the clinical potential of such immunotherapies. However, accelerating drug development and facilitating clinical translation require the convergence of interdisciplinary fields, AI, multi-omics, and nanomedicine. Therefore, integrating AI for predictive modeling, spatial omics for high-resolution analysis, biomarker-guided precise stratification, and combinatorial nanomedicine platforms for targeted delivery represents a core approach to targeting TAMs. This strategy promises to optimize treatment protocols, accelerate the clinical translation of gynecologic cancer therapies, and enhance therapeutic success rates.

## 8 Conclusion

In summary, within gynecological malignancies, the tumor-suppressing or tumor-promoting functions of TAMs are governed by microenvironmental signaling pathways, thereby making them promising therapeutic targets due to their dynamic nature. NDDSs demonstrate immense potential in the targeted modulation of TAM polarization, holding promise as a breakthrough approach for cancer immunotherapy. However, research regarding drugs targeting TAMs and NDDSs remains in its infancy. Advancing clinical translation will require the integration of multi-omics technologies, optimization of experimental models, and interdisciplinary collaboration.

## References

[1] Bray F Laversanne M Sung H Ferlay J Siegel RL Soerjomataram I . Global cancer statistics 2022: Globocan estimates of incidence and mortality worldwide for 36 cancers in 185 countries. CA Cancer J Clin. (2024) 74:229–63. doi: 10.3322/caac.21834 38572751

[2] Altea-Manzano P Decker-Farrell A Janowitz T Erez A . Metabolic interplays between the tumour and the host shape the tumour macroenvironment. Nat Rev Cancer. (2025) 25:274–92. doi: 10.1038/s41568-024-00786-4 39833533 PMC12140436

[3] Imani S Farghadani R Roozitalab G Maghsoudloo M Emadi M Moradi A . Reprogramming the breast tumor immune microenvironment: Cold-to-hot transition for enhanced immunotherapy. J Exp Clin Cancer Res. (2025) 44:131. doi: 10.1186/s13046-025-03394-8 40281554 PMC12032666

[4] Tufail M Jiang CH Li N . Immune evasion in cancer: Mechanisms and cutting-edge therapeutic approaches. Signal Transduct Target Ther. (2025) 10:227. doi: 10.1038/s41392-025-02280-1 40739089 PMC12311175

[5] Ghisoni E Morotti M Sarivalasis A Grimm AJ Kandalaft L Laniti DD . Immunotherapy for ovarian cancer: Towards a tailored immunophenotype-based approach. Nat Rev Clin Oncol. (2024) 21:801–17. doi: 10.1038/s41571-024-00937-4 39232212

[6] Corr BR Erickson BK Barber EL Fisher CM Slomovitz B . Advances in the management of endometrial cancer. BMJ. (2025) 388:e080978. doi: 10.1136/bmj-2024-080978 40044230

[7] Vitale I Manic G Coussens LM Kroemer G Galluzzi L . Macrophages and metabolism in the tumor microenvironment. Cell Metab. (2019) 30:36–50. doi: 10.1016/j.cmet.2019.06.001 31269428

[8] Li M Yang Y Xiong L Jiang P Wang J Li C . Metabolism, metabolites, and macrophages in cancer. J Hematol Oncol. (2023) 16:80. doi: 10.1186/s13045-023-01478-6 37491279 PMC10367370

[9] Bai X Guo YR Zhao ZM Li XY Dai DQ Zhang JK . Macrophage polarization in cancer and beyond: From inflammatory signaling pathways to potential therapeutic strategies. Cancer Lett. (2025) 625:217772. doi: 10.1016/j.canlet.2025.217772 40324582

[10] Xu J Ding L Mei J Hu Y Kong X Dai S . Dual roles and therapeutic targeting of tumor-associated macrophages in tumor microenvironments. Signal Transduct Target Ther. (2025) 10:268. doi: 10.1038/s41392-025-02325-5 40850976 PMC12375796

[11] Sun X Park MD Merad M Brown BD . Macrophages: Targets for next-generation cancer immunotherapy. Cancer Cell. (2026) 44:498–518. doi: 10.1016/j.ccell.2026.01.020 41759521 PMC13331334

[12] Karimova AF Khalitova AR Suezov R Markov N Mukhamedshina Y Rizvanov AA . Immunometabolism of tumor-associated macrophages: A therapeutic perspective. Eur J Cancer. (2025) 220:115332. doi: 10.1016/j.ejca.2025.115332 40048925

[13] von Locquenghien M Zwicky P Xie K Jaitin DA Sheban F Yalin A . Macrophage-targeted immunocytokine leverages myeloid, T, and NK cell synergy for cancer immunotherapy. Cell. (2025) 188:7099–117:e26. doi: 10.1016/j.cell.2025.10.030 41265436

[14] Pietrobono S De Vita V Mangiameli D Aparo A Lorenzo ES Bonato A . Egfr ligand angiogenin predicts response to ALK5 inhibition in pancreatic cancer via a TNF-alpha paracrine axis in tumor-associated macrophages. Oncogene. (2026) 45(20):1901–13. doi: 10.1038/s41388-026-03774-0 41975075 PMC13167465

[15] Fierro J DiPasquale J Perez J Chin B Chokpapone Y Tran AM . Dual-sgRNA CRISPR/Cas9 knockout of PD-L1 in human U87 glioblastoma tumor cells inhibits proliferation, invasion, and tumor-associated macrophage polarization. Sci Rep. (2022) 12:2417. doi: 10.1038/s41598-022-06430-1 35165339 PMC8844083

[16] Shmakova E Sudarskikh T Shalygina K Chagovets V Starodubtseva N Tokareva A . Inhibition of PFKFB3 in macrophages has a dual effect on tumor-regulating lipid metabolism. Int J Mol Sci. (2025) 27(1):217. doi: 10.3390/ijms27010217 41516095 PMC12785469

[17] Pang L Zhou F Liu Y Ali H Khan F Heimberger AB . Epigenetic regulation of tumor immunity. J Clin Invest. (2024) 134(12):e178540. doi: 10.1172/JCI178540 39133578 PMC11178542

[18] Grippin AJ Lee D Parkes EE Jiang W Kim BYS . Nanotechnology for immuno-oncology. Nat Cancer. (2025) 6:1311–25. doi: 10.1038/s43018-025-01025-x 40775548 PMC12412309

[19] Guan F Wang R Yi Z Luo P Liu W Xie Y . Tissue macrophages: Origin, heterogenity, biological functions, diseases and therapeutic targets. Signal Transduct Target Ther. (2025) 10:93. doi: 10.1038/s41392-025-02124-y 40055311 PMC11889221

[20] Zhao J Andreev I Silva HM . Resident tissue macrophages: Key coordinators of tissue homeostasis beyond immunity. Sci Immunol. (2024) 9:eadd1967. doi: 10.1126/sciimmunol.add1967 38608039

[21] Christofides A Strauss L Yeo A Cao C Charest A Boussiotis VA . The complex role of tumor-infiltrating macrophages. Nat Immunol. (2022) 23:1148–56. doi: 10.1038/s41590-022-01267-2 35879449 PMC10754321

[22] Zhang X Ji L Li MO . Control of tumor-associated macrophage responses by nutrient acquisition and metabolism. Immunity. (2023) 56:14–31. doi: 10.1016/j.immuni.2022.12.003 36630912 PMC9839308

[23] Wang Q Yu Y Ruan L Huang M Chen W Sun X . Integrated single-cell and bulk transcriptomic analysis identifies a novel macrophage subtype associated with poor prognosis in breast cancer. Cancer Cell Int. (2025) 25:119. doi: 10.1186/s12935-025-03750-w 40148933 PMC11948682

[24] Ma RY Black A Qian BZ . Macrophage diversity in cancer revisited in the era of single-cell omics. Trends Immunol. (2022) 43:546–63. doi: 10.1016/j.it.2022.04.008 35690521

[25] Nasir I McGuinness C Poh AR Ernst M Darcy PK Britt KL . Tumor macrophage functional heterogeneity can inform the development of novel cancer therapies. Trends Immunol. (2023) 44:971–85. doi: 10.1016/j.it.2023.10.007 37995659

[26] Xu Y Li S Wu J Xu S Shen M Wang C . Targeting cystine metabolism in the lung cancer environment enhances the efficacy of immune checkpoint inhibition. Adv Sci (Weinh). (2025) 12:e13084. doi: 10.1002/advs.202413084 40637137 PMC12463131

[27] Tang B Zhu J Wang Y Chen W Fang S Mao W . Targeted XCT-mediated ferroptosis and protumoral polarization of macrophages is effective against HCC and enhances the efficacy of the anti-PD-1/L1 response. Adv Sci (Weinh). (2023) 10:e2203973. doi: 10.1002/advs.202203973 36442849 PMC9839855

[28] Scott CL Banerjee S Joly F Lee JM Mukhopadhyay A Tan DS . Ovarian cancer. Nat Rev Dis Primers. (2026) 12(1):10. doi: 10.1038/s41572-026-00686-x 41786760

[29] Ghisoni E Benedetti F Minasyan A Desbuisson M Cunnea P Grimm AJ . Myeloid cell networks govern re-establishment of original immune landscapes in recurrent ovarian cancer. Cancer Cell. (2025) 43:1568–86:e10. doi: 10.1016/j.ccell.2025.07.005 40749672 PMC12933722

[30] Yan Y Lu J Luo H Wang Z Xu K Wang L . Decoding immune low-response states in ovarian cancer: Insights from single-cell and spatial transcriptomics for precision immunotherapy. Front Immunol. (2025) 16:1667464. doi: 10.3389/fimmu.2025.1667464 41050676 PMC12491270

[31] Osborn G Lopez-Abente J Adams R Laddach R Grandits M Bax HJ . Hyperinflammatory repolarisation of ovarian cancer patient macrophages by anti-tumour IgE antibody, MOV18, restricts an immunosuppressive macrophage:Treg cell interaction. Nat Commun. (2025) 16:2903. doi: 10.1038/s41467-025-57870-y 40210642 PMC11985905

[32] Li J Lu J Zheng C Huang X Li H Mai Q . Serotonin-licensed macrophages potentiate chemoresistance via inositol metabolic crosstalk in ovarian cancer. Cell Metab. (2026) 38:331–49:e10. doi: 10.1016/j.cmet.2025.11.011 41412121

[33] Liu Y Jiang LJ Liu HF Chen L Guo L Ge J . Distinct roles of HMOX1 on tumor epithelium and macrophage for regulation of immune microenvironment in ovarian cancer. Int J Surg. (2025) 111:6725–42. doi: 10.1097/JS9.0000000000002829 40638251 PMC12527711

[34] Yin B Ding J Liu J Hu H Zhu Y Yang M . Exosomal CMTM4 induces immunosuppressive macrophages to promote ovarian cancer progression and attenuate anti-PD-1 immunotherapy. Adv Sci (Weinh). (2025) 12:e04436. doi: 10.1002/advs.202504436 40433989 PMC12376563

[35] Mollaoglu G Tepper A Falcomata C Potak HT Pia L Amabile A . Ovarian cancer-derived IL-4 promotes immunotherapy resistance. Cell. (2024) 187:7492–510:e22. doi: 10.1016/j.cell.2024.10.006 39481380 PMC11682930

[36] Qiu JG Sang P Zhou FM Zhang XQ Wang W Qian YC . Novel role of MKRN2 in regulating tumor growth through host microenvironment and macrophage M1 to M2 switch. Cancer Lett. (2025) 633:218035. doi: 10.1016/j.canlet.2025.218035 40925500

[37] Wang G Huang S Yin B Shangguan F Ma J Li A . SLC40A1-mediated positive feedback loop with M1 macrophages suppresses epithelial ovarian cancer progression. Front Immunol. (2025) 16:1709597. doi: 10.3389/fimmu.2025.1709597 41613134 PMC12847247

[38] Elorbany S Berlato C Carnevalli LS Maniati E Barry ST Wang J . Immunotherapy that improves response to chemotherapy in high-grade serous ovarian cancer. Nat Commun. (2024) 15:10144. doi: 10.1038/s41467-024-54295-x 39578450 PMC11584700

[39] Wang Y He M Zhang C Cao K Zhang G Yang M . Siglec-9(+) tumor-associated macrophages delineate an immunosuppressive subset with therapeutic vulnerability in patients with high-grade serous ovarian cancer. J Immunother Cancer. (2023) 11(9):e007099. doi: 10.1136/jitc-2023-007099 37709296 PMC10503378

[40] Yu F Zhou Q Yu W Zhou T Cao C Xie Y . Tumor-associated macrophages promote chemoresistance to paclitaxel via activating Notch2-Jag1 juxtacrine signaling. Mol Cancer. (2026) 25(1):135. doi: 10.1186/s12943-025-02546-w 41519773 PMC13191955

[41] Tong Y Gao Y Wu X Kang Y Zheng H Yu R . Differential mediating roles of immune-inflammatory cells in ≥HSIL women: A focus on HPV and CD4/CD8 or NLR. J Inflammation Res. (2025) 18:16699–712. doi: 10.2147/JIR.S565862 41341213 PMC12669056

[42] Liu Y Li L Li Y Zhao X . Research progress on tumor-associated macrophages and inflammation in cervical cancer. BioMed Res Int. (2020) 2020:6842963. doi: 10.1155/2020/6842963 32083131 PMC7011341

[43] Xia P Zhou J Shen R Wang D . Deciphering the cellular and molecular landscape of cervical cancer progression through single-cell and spatial transcriptomics. NPJ Precis Oncol. (2025) 9:158. doi: 10.1038/s41698-025-00948-z 40437003 PMC12120119

[44] Chen F Bai G Liu Q He G Ding Z Liang J . Integrated multi-omics identifies a CD54(+) iCAF-ITGAL(+) macrophage niche driving immunosuppression via CXCL8-PD-L1 axis in cervical cancer. Mol Cancer. (2025) 24:262. doi: 10.1186/s12943-025-02471-y 41121145 PMC12539134

[45] Chen XJ Wei WF Wang ZC Wang N Guo CH Zhou CF . A novel lymphatic pattern promotes metastasis of cervical cancer in a hypoxic tumour-associated macrophage-dependent manner. Angiogenesis. (2021) 24:549–65. doi: 10.1007/s10456-020-09766-2 33484377 PMC8292274

[46] Dang T You Y Wei L Li Q Sun H Sun M . ICAT drives lactylation of tumor-associated macrophages via the c-Myc-ENO1 axis to promote cervical cancer progression. Free Radic Biol Med. (2025) 241:316–29. doi: 10.1016/j.freeradbiomed.2025.09.031 40976412

[47] Sun Y Zhang J Guo L Zhang J Chen Q Zhang T . Discovering the abnormalities and functional importance of ferroptosis-related molecules in cervical cancer. Int J Med Sci. (2026) 23:443–60. doi: 10.7150/ijms.107133 41583517 PMC12825119

[48] Wang Q Sudan K Schmoeckel E Kost BP Kuhn C Vattai A . CCL22-polarized TAMs to M2a macrophages in cervical cancer *in vitro* model. Cells. (2022) 11(13):2027. doi: 10.3390/cells11132027 35805111 PMC9265611

[49] Yin B Chen C Huang B Ding J Hu H Zhou H . Oncogenic CMTM6 drives M2a macrophages formation and fuels cervical cancer progression. Front Immunol. (2025) 16:1621816. doi: 10.3389/fimmu.2025.1621816 40761779 PMC12319053

[50] Yi S Hu S Li W Cai J Cai L Wang L . Knocking down hypoxia-induced semaphorin 6B may attenuate the progression of cervical cancer through regulating macrophage M2 polarization. J Transl Med. (2025) 23:776. doi: 10.1186/s12967-025-06692-z 40640851 PMC12247440

[51] Wang Z Wang B Feng Y Ye J Mao Z Zhang T . Targeting tumor-associated macrophage-derived Cd74 improves efficacy of neoadjuvant chemotherapy in combination with Pd-1 blockade for cervical cancer. J Immunother Cancer. (2024) 12(8):e009024. doi: 10.1136/jitc-2024-009024 39107132 PMC11308911

[52] Yang Y Tian X Chen J Liu J Jiang H Liu L . Targeting Hk3 in tumor-associated macrophages enhances antitumor immunity through augmenting antigen cross-presentation in cervical cancer. J Immunother Cancer. (2025) 13(7):e011948. doi: 10.1136/jitc-2025-011948 40664447 PMC12265828

[53] How JA Jazaeri AA Westin SN Lawson BC Klopp AH Soliman PT . Translating biological insights into improved management of endometrial cancer. Nat Rev Clin Oncol. (2024) 21:781–800. doi: 10.1038/s41571-024-00934-7 39198622 PMC12084113

[54] Foley KG Adli M Kim JJ . Single-nuclei sequencing of uterine serous carcinoma reveals racial differences in immune signaling. Proc Natl Acad Sci USA. (2024) 121:e2402998121. doi: 10.1073/pnas.2402998121 39133838 PMC11348309

[55] Ye J Yan Y Sun X Wu Q Shi Y Zhang M . Targeting the Cd47-Hck-Lgals9 axis disrupts proliferation-immunosuppression coupling in early-stage endometrial cancer. Mol Cancer. (2025) 25(1):61. doi: 10.1186/s12943-025-02534-0 41437376 PMC12969882

[56] Ye L Jiang G Sun Y Li B . Arntl-mediated Ino80-Dhx15 axis reprograms the glycolytic metabolism and augments the progression of endometrial carcinoma. Cell Death Dis. (2025) 16:463. doi: 10.1038/s41419-025-07776-w 40541951 PMC12181345

[57] Bernhard WF Jones JE Friedberg DZ Litwin SB . Ascending aorta-right pulmonary artery shunt in infants and older patients with certain types of cyanotic congenital heart disease. Circulation. (1971) 43:580–4. doi: 10.1161/01.cir.43.4.580 4102252

[58] Liu X Sun H Liang J Yu H Xue M Li Y . Metabolic interplay between endometrial cancer and tumor-associated macrophages: Lactate-induced M2 polarization enhances tumor progression. J Transl Med. (2025) 23:923. doi: 10.1186/s12967-025-06235-6 40826409 PMC12359828

[59] Yang S Ma Y Wu T Huang X . Lactate transmission from hypoxic tumor cells promotes macrophage senescence and M2 polarization via the Dnmt1-Nhe7 axis to accelerate endometrial cancer progression. Cell Death Dis. (2026) 17:185. doi: 10.1038/s41419-026-08411-y 41617670 PMC12876988

[60] Pan X Wang W Wang Y Gu J Ma X . M2 macrophage-secreted Kynu promotes stemness remodeling and Malignant behavior in endometrial cancer via the Sod2-Mtros-Ero1alpha-Upr(Er) axis. J Exp Clin Cancer Res. (2025) 44:193. doi: 10.1186/s13046-025-03285-y 40616124 PMC12231660

[61] Haber T Cornejo YR Aramburo S Flores L Cao P Liu A . Specific targeting of ovarian tumor-associated macrophages by large, anionic nanoparticles. Proc Natl Acad Sci USA. (2020) 117:19737–45. doi: 10.1073/pnas.1917424117 32732430 PMC7443897

[62] Pires IS Covarrubias G Gomerdinger VF Backlund C Nombera Bueno E Billingsley MM . Il-12-releasing nanoparticles for effective immunotherapy of metastatic ovarian cancer. Nat Mater. (2026) 25:322–34. doi: 10.1038/s41563-025-02390-9 41174039 PMC12867765

[63] Zhang L Xu C Li M Lu X Sheng Y Chen L . Gambogic acid based coordination polymer reinforces high-intensity focused ultrasound treatment of gynecologic Malignancies. Adv Mater. (2025) 37:e2501664. doi: 10.1002/adma.202501664 40223396

[64] Luo M Zhao FK Ouyang CH Wang YM Luo Y Bian J . Recent advances in nanomaterial-based precision medicine for orthotopic tumor therapy. J Nanobiotechnology. (2026) 24:168. doi: 10.1186/s12951-026-04056-3 41580747 PMC12910877

[65] Gomerdinger VF Nabar N Hammond PT . Advancing engineering design strategies for targeted cancer nanomedicine. Nat Rev Cancer. (2025) 25:657–83. doi: 10.1038/s41568-025-00847-2 40751005

[66] Sabit H Pawlik TM Radwan F Abdel-Hakeem M Abdel-Ghany S Wadan AS . Precision nanomedicine: Navigating the tumor microenvironment for enhanced cancer immunotherapy and targeted drug delivery. Mol Cancer. (2025) 24:160. doi: 10.1186/s12943-025-02357-z 40457437 PMC12131435

[67] Li L Shan T Zhang D Ma F . Nowcasting and forecasting global aging and cancer burden: Analysis of data from the Globocan and Global Burden of Disease Study. J Natl Cancer Cent. (2024) 4:223–32. doi: 10.1016/j.jncc.2024.05.002 39281725 PMC11401500

[68] Zhao X Ran J Li S Chen J . Advances and obstacles of T cell-based immunotherapy in gynecological Malignancies. Mol Cancer. (2025) 24:207. doi: 10.1186/s12943-025-02411-w 40713697 PMC12297529

[69] Strizova Z Benesova I Bartolini R Novysedlak R Cecrdlova E Foley LK . M1/M2 macrophages and their overlaps - myth or reality? Clin Sci (Lond). (2023) 137:1067–93. doi: 10.1042/CS20220531 37530555 PMC10407193

[70] Jin R Neufeld L McGaha TL . Linking macrophage metabolism to function in the tumor microenvironment. Nat Cancer. (2025) 6:239–52. doi: 10.1038/s43018-025-00909-2 39962208

[71] Wang X Zhang S Xue D Neculai D Zhang J . Metabolic reprogramming of macrophages in cancer therapy. Trends Endocrinol Metab. (2025) 36:660–76. doi: 10.1016/j.tem.2024.08.009 39304355

[72] Suarez-Carmona M Hampel M Zhang XW Pochmann A Grauling-Halama SA Valous NA . Harnessing lipid-driven immunometabolic pathways in omental metastases to enhance immunotherapy in patients with ovarian cancer. Signal Transduct Target Ther. (2026) 11(1):78. doi: 10.1038/s41392-026-02594-8 41775684 PMC12957430

[73] Alradwan I Zhi P Zetrini AE Wang L He C Rezaeifarimani M . Empowering chemotherapy-induced antitumor immunity by multi-targeted synergistic combination nanomedicine for triple-negative breast cancer. Mater Today Bio. (2025) 35:102445. doi: 10.1016/j.mtbio.2025.102445 41255415 PMC12621471

[74] Chen S Zhang P Zhu G Wang B Cai J Song L . Targeting Gsdme-mediated macrophage polarization for enhanced antitumor immunity in hepatocellular carcinoma. Cell Mol Immunol. (2024) 21:1505–21. doi: 10.1038/s41423-024-01231-0 39496854 PMC11607431

[75] Ren K Yuan Z Lei L Xiao Z Ma N Wang G . Engineered Bcg selectively triggers trained immunity in tumor-associated macrophages and sensitizes glioblastoma to radiotherapy in mice. Nat Commun. (2026). doi: 10.1038/s41467-026-72067-7 42009673 PMC13284352

